# Assessment of trivalent live influenza vaccines in MDCK cell line

**DOI:** 10.1016/j.mex.2021.101442

**Published:** 2021-07-04

**Authors:** G. Landgraf, Y.A. Desheva, L.G. Rudenko

**Affiliations:** a Federal State Budgetary Educational Institution of Higher Professional Education "St. Petersburg State University", St. Petersburg, Russian Federation; b Federal State Budget Scientific Institution "Institute of Experimental Medicine", St. Petersburg, Russian Federation

**Keywords:** Live influenza vaccine, Virus replication, TaqMan technique

## Abstract

We applied a one-step reverse transcriptase real-time PCR (rRT-PCR) analysis using TaqMan technique to evaluate the infectious titers of vaccine strains containing in trivalent live influenza vaccines (LAIVs).

The cold-adapted reassortant influenza viruses A/H1N1 pdm09, A/H3N2, B/Yamagata and B/Victoria, included in the composition of the LAIV in 2015-2016, 2017-2018 and 2018-2019 flu season were studied for reproductive activity in MDCK cells as part of a mono-vaccine and tri-vaccine. For this we have developed a set of specific primers and probes. Method validation was performed using ELISA-test after mouse monoclonal antibodies to hemagglutinin (HA) staining of MDCK monolayer.

Influenza B viruses B/Yamagata and B/Victoria were studied in MDCK cells in mono-infection and coinfection with different multiplicity of infection (MOI) using quantitative rRT-PCR.

• RT-PCR analysis was adjusted to assess the growth characteristics of cold-adapted reassortant influenza viruses in MDCK cell line. The greatest suppression in the composition of the tri-vaccine was exposed to the H1N1 pdm09 LAIV component.
• Influenza B viruses are least suppressed in trivalent LAIV. Influenza viruses B/Yamagata and B/Victoria reproduced as part of a mixed preparation not lower, if not better than as a mono-preparation at an MOI of 0.1. At an MOI of 0.01, the reproduction of both B/Yamagata and B/Victoria in the mixture was reduced compared to mono-vaccine.
• The interference of trivalent LAIV vaccine viruses in MDCK cells was minimal at low dilutions. This indicates that it is undesirable to reduce the titers of vaccine viruses, including at the stages of transportation and storage of LAIV

## Graphical abstract

**Figure:**
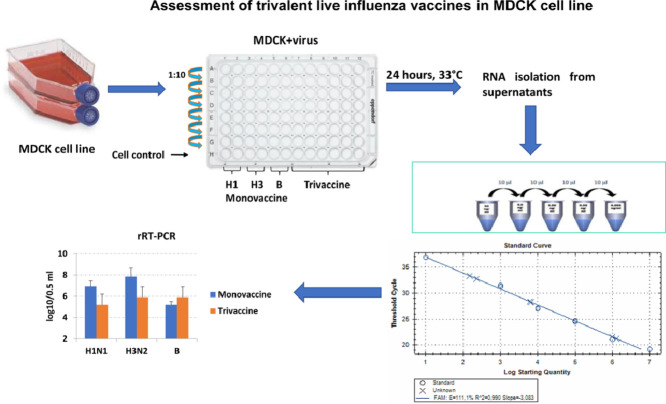

Specifications table

**Table:** 

Subject Area:	Immunology and Microbiology
More specific subject area:	Virology
Method name:	Titration of three live influenza vaccine viruses in MDCK cell line using TaqMan technique
Name and reference of original method:	Zang Y, Du D, Ge P, Xu Y, Liu X, Zhang Y, Su W, Kiseleva I, Rudenko L, Xu F, Kong W, Development of one-step real-time PCR assay for titrating trivalent live attenuated influenza vaccines, Human vaccines & immunotherapeutics, 2 (2014) 3642-8, doi:10.4161/hv.34453 Shcherbik S, Sergent SB, Davis WG, Shu B, Barnes J, Kiseleva I, Larionova N, Klimov A, Bousse T, Application of real time RT-PCR for the genetic homogeneity and stability tests of the seed candidates for live attenuated influenza vaccine production. Journal of virological methods, 1(2014) 18-25, doi: 10.1016/j.jviromet.2013.09.003 Biere B, Bauer B, Schweiger B. Differentiation of influenza B virus lineages Yamagata and Victoria by real-time PCR. Journal of clinical microbiology. 2010 Apr 1;48(4):1425-7. doi: 10.1128/JCM.02116-09
Resource availability:	Reagents and conditions necessary for reproducing the method is provided in the text of the article

## Method details

### Background

The method of one-step reverse transcriptase real-time PCR (rRT-PCR) is widely applied in detecting influenza viruses [1,2]. The seasonal trivalent live attenuated influenza vaccines (LAIVs) include three vaccine strains: A/H1N1, A/H3N2 and one influenza B virus based on cold-adapted master donor strain. In Russia, a cold-adapted master donor strain (MDS) A/Leningrad/134/57 (H2N2) is currently used to prepare the reassortant viruses A/H1N1 and A/H3N2 [3]. For the production of vaccine strains of type B influenza viruses, the MDS B/USSR/60/69 was developed [4]. The quadrivalent LAIV formulation comprises two influenza B viruses belonging to B/Yamagata or B/Victoria antigenic lineages [5]. We have applied a TaqMan rRT-PCR technique for trivalent LAIVs titration in MDCK cell line.

The study used reassortant vaccine strains of influenza A and B viruses from the collection of the Virology Department of the Federal State Budget Scientific Institution "Institute of Experimental Medicine". The monovalent vaccine strains (A/H1N1 pdm09, A/H3N2 and B) were prepared based on the WHO recommended epidemic viruses for 2015-2016, 2017-2018 and 2018-2019 influenza seasons. The names of vaccine viruses and corresponding epidemic strains recommended by WHO for the preparation of influenza vaccines are presented in Table 1. All viruses were propagated in 10-day-old developing embryonated chicken eggs (CE). The infectious activity of viruses was determined in CE at the temperature optimal for influenza A and B viruses (33°C), the 50% embryonic infectious dose (EID_50_) was calculated using the Reed and Muench method [6]. The last column of Table 1 shows the EID_50_ titers of monovalent vaccine strains (A/H1N1, A/H3N2 and B) indicated in the passport of the vaccine.

**Table 1:** The composition of vaccine viruses included in the trivalent LAIV

Flu season	Serotype	Strain name	Name of the WHO recommended CVV strains	Virus titer (lоg10 EID_50_/0.5 ml)
2015-2016	H1N1 pdm09	A/17/ California /2009/38	A/17/California/2009/38 (H1N1)pdm09	7.4±0.4
	H3N2	A/17/ Switzerland /2013/1	A/17/ Switzerland /2013/1 (H3N2)	9.1±0.5
	B	B/60/ Phuket /2013/26 (Yamagata)	B/60/ Phuket /2013/26 (Yamagata lineage)	8.9±0.9
2017-2018	H1N1 pdm09	A/17/ New York /2015/5364	A/17/ New York /2015/5364 (H1N1) pdm09	8.4±0.1
	H3N2	A/17/ Hong Kong /2014/8296	A/17/Hongkong/2014/8296 (H3N2)	8.0±0.3
	B	B/60/ Brisbane /2008/83 (Victoria)	B/60/Brisbane/2008/83 (Victoria lineage)	7.9±0.3
2018-2019	H1N1 pdm09	A/17/ New York /2015/5364	A/17/New York/2015/5364 (H1N1)	8.2±0.2
	H3N2	А/17/Singapore/2016/3571	А/17/Singapore/2016/3571 (H3N2)	8.1±0.1
	В	В/60/Colorado/2017/1 (Victoria)	В/60/Colorado/2017/1 (Victoria lineage)	8.2±0.3

## Procedure

### Infection of the MDCK cell line

The MDCK NBL-2 cell line was obtained from the Center for Disease Control and Prevention (Atlanta, GA, USA).

The reassortant influenza viruses were titrated onto a monolayer of cells in 96-well plates by introducing a series of 10-fold dilutions of viruses (A/H1N1, A/H3N2, B or trivalent LAIV, respectively) in DMEM (supplemented with trypsin TPCK at a concentration of 2 μg/ml) in volume 100 μl. In order to obtain trivalent vaccine composition, the corresponding vaccine strains grown in CE were combined into a joint preparation so that the dose of each vaccine virus per 0.5 ml matches the value indicated in the Table 1.

**Fig. 1:**
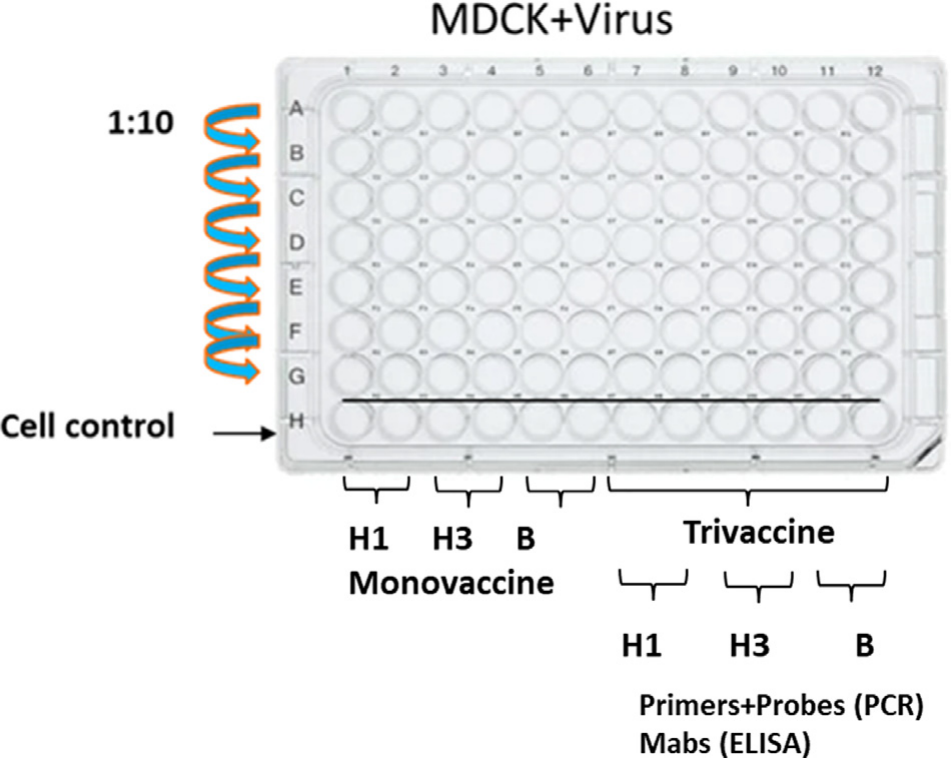
Titration of viruses in MDCK cells by decreasing 10-fold dilutions.

The experimental setup is shown in Fig. 1. All experiments were carried out in at least 2 repetitions.

After one hour of incubation at 33°C in an atmosphere with 5% CO_2_, the supernatants were removed by careful pipetting, washed by PBS and 100 μl of supporting medium (DMEM supplemented with trypsin TPCK at a concentration of 2 μg/ml) was added to the wells of the plate. The infected culture was incubated in a CO_2_ atmosphere at an optimum temperature of 33°C for 24 h, monitoring the state of the monolayer under a microscope. The culture supernatants were used for subsequent rRT-PCR.

*The one-*step *real-time polymerase chain reaction with reverse transcription*

Before one-step rRT-PCR, the viral RNA was isolated from 80 μl of virus-containing culture supernatants using the QIAamp Viral RNA Mini Kit (Qiagen, Hulsterweg, Netherlands) according to manufacturer's instructions.

We have developed a set of specific primers and probes using Beacon Designer software (Thermo Fisher Scientific, Waltham, MA USA). The sequences of primers and probes are shown in Table 2.

**Table 2:** Primers and probes for polyvalent LAIV components, developed using a computer program Beacon Designer.

Virus	Gene	Name of primers and probes	Sequence of primers and probes (5′ - 3′)
A/17/California/2009/38 (H1N1)pdm09	НА	F-677	AGTTCAAGCCGGAAATAGCAAT
		R-845	ATACCAGATCCAGCATTTCTTTC
		Pr-704F	(FAМ) - CAAAGTGAGGGATCAAGAAGGGAGAAT - BHQ-1
A/17/ New York/2015/5364 (H1N1) pdm09	НА	F-853	F- GCTGGATCTGGTATTATC
		R-963	R- TTCAGAATATACATCCGATCACAATTGG
		Probe-874F	(FAМ) - CAGATACACCAGTCCACGATTGC- BHQ-1
A/17/ Switzerland /2013/1 (H3N2)	НА	F -65	TCTGGTTTTCGCTCAAAA
		R -177	TTCGGTCATTCGTGATTG
		Pr-95F	(FAM) - TGACAATAGCACGGCAACGC -BHQ-1
А/17/Singapore/2016/3571 (H3N2)	НА	F-203	GGTTCAGAATTCCTCAATAG
		R-385	GGCACATCATAAGGGTAA
		Pr-231F	(FAM) - TGCGACAGTCCTCATCAGATCC- BHQ-1
В/USSR/60/69	NP	F-233	AAGCTGATGTCGGAAGGAGA
		R-402	TTCCACAGCATGTGCATTTT
		Pr-253F	(ROX) - ACCCAAAAGAAACAAACCCC-BHQ-2
B/60/Brisbane/2008/83 (Victoria lineage)	HA	F-741	CACACATTACGTTTCACAG
		R- 835	GCACCATGTAATCAACAAC
		Pr – 780F	(FAM) - AACAGAAGACGGAGGACTACCAC - BHQ-1
Influenza B viruses	HA	F 432	ACCCTACARAMTTGGAACYTCAGG
		R 479	ACAGCCCAAGCCATTGTTG
B/Victoria	HA	Pr 470 R	(CY5) - AAATCCCGTTTCCATTGGTAATGT BHQ2
B/Yamaga	HA	Pr 437F	(ROX) - AATCCGMTYTTACTGGTAG BHQ2

One-step rRT-PCR based on the TaqMan technology was performed using the SuperScript kit III Platinum One-Step Quantitative RT-PCR (Invitrogen, Grand Island, NY, USA) on a CFX96 thermocycler (Bio-Rad, Hercules, CA, USA) according to the manufacturer's instructions. The total volume of the reaction mixture was 25 μl. Virus titers were determined as the last dilution, which gave a positive result of RT-PCR and expressed in log 10. In each experiment, at least 2 biological repeats (at least two independent experiments, in some cases it was three independent experiments) were performed; the RT-PCR analysis was performed in duplicates.

### ELISA with a fixated MDCK monolayer

The remaining viral monolayer was fixated with 80% acetone in phosphate buffered saline (PBS). The HA expression on the cell surface was studied in ELISA-test with monoclonal antibodies (MAbs), as described below. The fixated cell monolayer was washed 4 times with phosphate buffered saline (PBS) contained 0.05% Tween-20 and treated with MAbs at a concentration of 0.1 μg / 1 ml (150 µl/well). The mouse MAbs to HA of influenza H1 and H3 viruses and NP protein of influenza B viruses used in this study are presented in Table 3.

**Table 3:** Monoclonal antibodies used for ELISA

Influenza virus subtype	Monoclonal antibody
Н1	Anti-Swine H1N1-HA mAb, catalog #10033-3f3, A10MOOGA90 15973 (Abnova, Taiwan Taipei)
Н3	Anti H3 (H3N2) mAb, clone In A246, catalog #MAB 1271, Lot # HA161, B1ENO1040F00009 (Abnova, Taiwan Taipei)
В	MAB8259, Mouse Anti Influenza B Monoclonal Antibody, lot 2817707, # 56005 (Millipore Corp., USA)

ELISA was performed with goat anti mouse IgG antibodies labeled with horseradish peroxidase and TMB substrate, the reaction was stopped with 1N H_2_SO_4_. The expression of HA or B-NP in the infected cells was determined on a microplate reader (ELx800, Bio-Tek Instruments Inc, USA) at a wavelength of 450 nm. The threshold value for determining the presence of the virus was the average optical density (OD) for three negative wells that did not contain the virus plus three standard deviations. A 50% tissue culture infectious dose (TCID_50_) was calculated according to the method of Reed and Muench [6].

*Quantitative analysis of influenza B/Yamagata and B/Victoria vaccine strains in MDCK cell line*

Co-infection with influenza viruses B/60/Wisconsin/ 2010/125 (Yamagata lineage) and B/60/Brisbane/2008/83 (Victoria lineage) was carried out on the formed MDCK monolayer in 24 well plates for cell cultures (10^5^ cells per 1 ml). Viruses were introduced with a multiplicity of infection (MOI) of 0.1 - 0.01/1 ml. To determine the multiplicity of infection, viruses were titrated in MDCK cells with determination of infectious titers by conventional methods as described elsewhere [7]. The supernatants were collected 24 h after infection. Viral RNA was isolated from the supernatant.

To construct a standard curve, we used serial dilutions of viral RNA isolated from vaccine viruses cultivated in CE with a known infectious titer EID50/ 0.5 ml in nuclease-free water (Invitrogen, Grand Island, NY, USA).

**Fig. 2:**
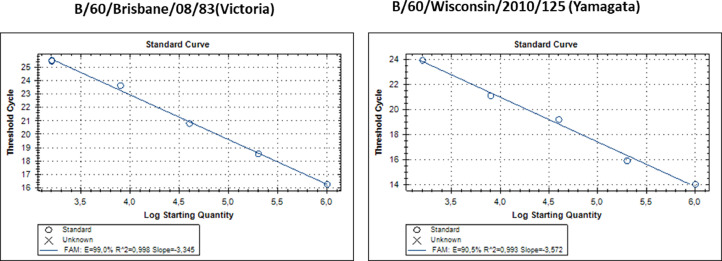
The standard curve constructed as the dependence of the Ct values from Log2 of the initial number of copies of the transcript of the standard viral material. Viral RNA was isolated from vaccine strains B/60/Brisbane/2008/83 (Victoria lineage) (8.0 EID_50_/0,5 ml) and B/60/Wisconsin/2010/125 (Yamagata lineage) (8.8 EID_50_/0,5 ml).

When an amplification efficiency consists of about 100%, the matrix doubling occurs in each amplification cycle. Therefore, a relative quantitative assessment of the content of viruses was determined by multiplying the concentration of the standard by the level of change in the expression of the HA gene according to the formula (1):

(1) $$C_{\left(\text{Sample}\right)}=C_{\text{Standard}}*2^{\left(\text{Ct}\left(S\tan dard\right)-\left(\text{Sample}\right)\right)}$$

where: C (Sample) - is the concentration of the sample; C (Standard) - is the concentration of the standard; Ct - is the threshold cycle. The results were expressed in log10. The results of determining the rRT-PCR efficiency and constructing a standard curve are presented in Fig. 2.

To determine the efficiency of the rRT-PCR reaction, at least five serial 5-fold dilutions of viral RNA were performed. When constructing a standard curve, the correlation coefficient (R2), plotted by plotting Ct values on 5-fold serial dilutions of a viral RNA sample containing 10^3^-10^6^ copies, showed values in the range of 0.99; the efficiency (E) of passing the reactions for each target ranged from 90.5 to 99%. These results confirmed the high specificity and efficiency of the assay.

### Statistical analysis

Statistical processing of the results was carried out using the Excel or GraphPad software (San Diego, CA, USA). To present the data, we used indicators of descriptive statistics: means and standard deviation. Comparisons of two independent groups was performed using the nonparametric Mann – Whitney test. Differences were considered statistically significant at p <0.05.

## Method validation

1. As shown in Fig. 3, a decrease in the reproduction of A/H1N1 pdm09 virus strains A/17/California/2009/38 (H1N1)pdm09 and A/17/New York/15/5364) in the MDCK cell culture in the trivalent vaccine compared to the corresponding mono-preparations by an average of 100 times and these differences were only 6 out of 18 differences are statistically significant when confirmed either by PCR or by ELISA with monoclonal antibodies.

**Fig. 3:**
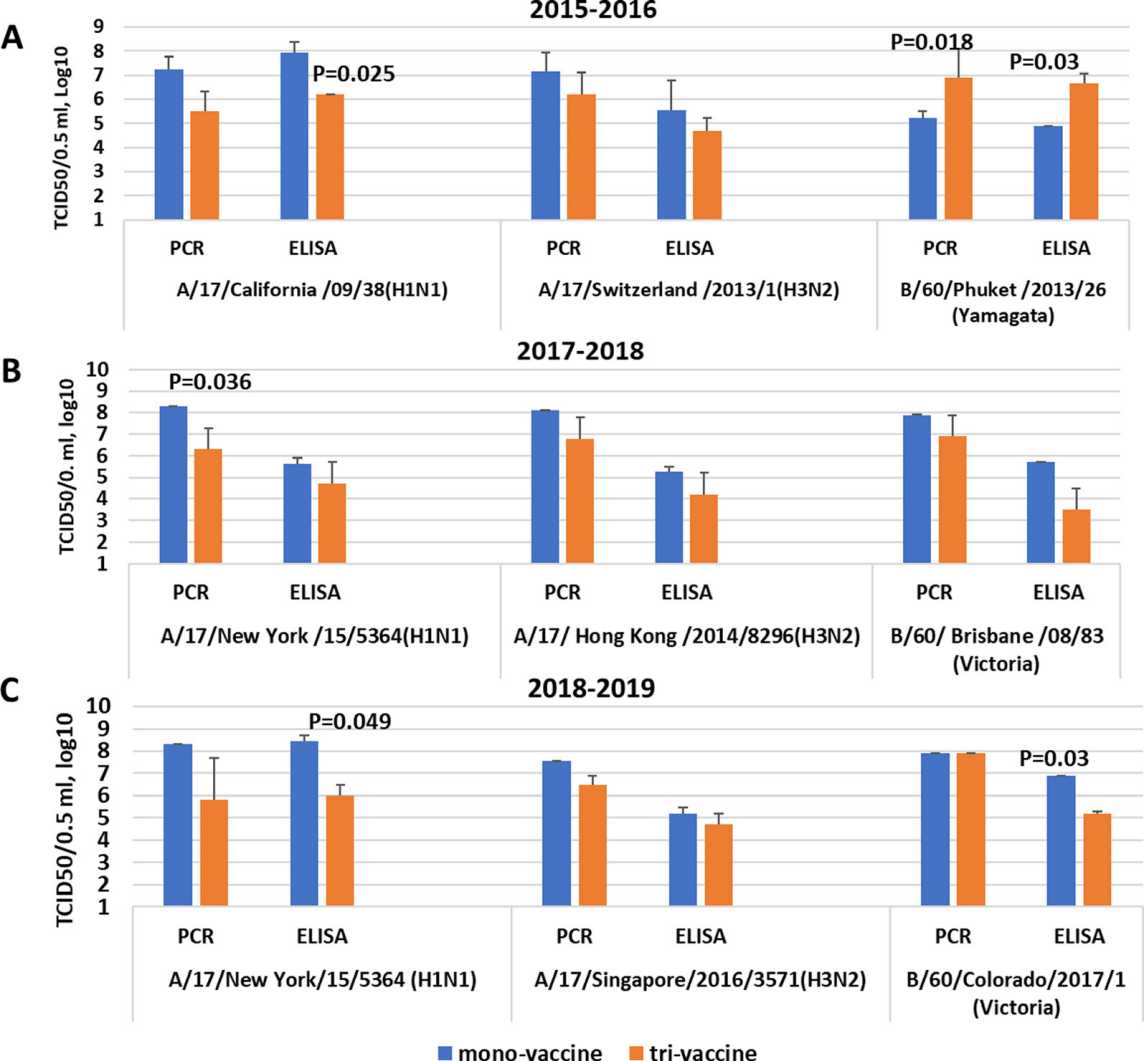
Replication of vaccine viruses in MDCK cells after infection with monovalent and trivalent LAIVs when evaluated by rRT-PCR or ELISA. A. Trivalent vaccine 2015-2016 years of formulation. When performing rRT-PCR, we used primers and probes for HA of influenza A viruses and NP B/USSR/60/69 influenza virus (see Table 2). B. Trivalent vaccine 2017-2018 years of formulation. Primers and probes for HA of influenza A and B/60/Brisbane/2008/83 (Victoria lineage) viruses were used (see Table 2). C. Trivalent vaccine 2018-2019 years of formulation. Primers and probes for HA of influenza A and B viruses were used (see Table 2).
2. The reprlication of influenza A/H3N2 strains (A/17/Switzerland /2013/1 (H3N2), A/17/Hong Kong/2014/8296 and А/17/Singapore/2016/3571 (H3N2) in the trivalent vaccine was reduced by 10-30 times compared with mono-preparations however, the differences were not statistically significant. Reproduction of the vaccine strain of the influenza virus of the Yamagata antigenic lineage (B/60/Phuket/2013/26) for the epidemic season 2015-2016 was even slightly higher in comparison with the mono-vaccine, and the reproduction of the vaccine strain B/60/Brisbane/2008/83 (Victoria lineage) and В/60/Colorado/2017/1 (Victoria lineage) in the composition of the trivalent vaccine was equal or decreased compared to mono-vaccine (Fig. 3). The more pronounced inhibition of the replication of influenza A viruses in the trivalent vaccine compared to influenza B viruses can be explained by the fact that influenza B viruses are able to suppress the multiplication of influenza A viruses in heterogeneous mixtures to some extent. Previously, it was shown in vitro that this may be the result of suppression of the synthesis of proteins HA and NP of influenza A virus at the stage of primary transcription [8].
3. Fig. 4 shows that minimal differences in the reproduction of influenza A vaccine viruses in the trivalent LAIV were observed at the lowest dilutions. With falling dilutions when the viral material was inoculated into MDCK cells, the difference in the number of copies in the trivalent preparations as compared with the mono-vaccine increased.

**Fig. 4:**
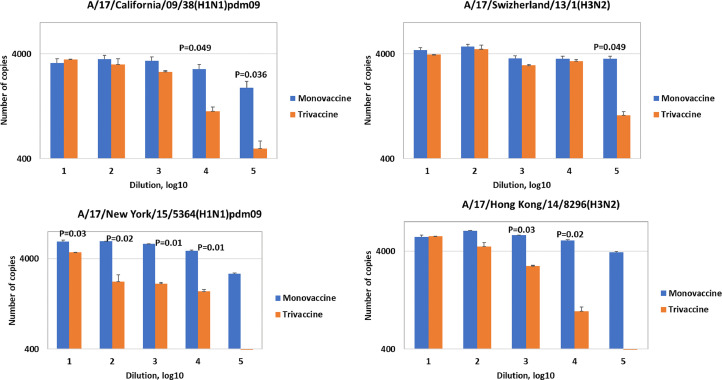
Determination of viral load in serial dilutions of mono-vaccines and trivalent LAIVs. The data from one of two representative experiments are presented. All rRT-PCR tests were performed in duplicates.
  These results show that the minimum reduction in the replication of vaccine strains in a trivaccine can be achieved with equal infectious doses of monovalent strains.
  When studying B/60/Wisconsin/2010/125 (Yamagata lineage) and B/60/Brisbane/2008/83 (Victoria lineage) influenza virus replication it wаs shown that both viruses B/Yamagata and B/Victoria reproduced as part of a mixed preparation not lower, if not better than as a mono-preparation at an MOI of 0.1 (Fig. 5). At an MOI of 0.01, the reproduction of both B/Yamagata and B/Victoria in the mixture was reduced compared to mono-vaccine. At the same time, the reproduction of the B/Victoria virus decreased to a greater extent (P=0.02).

**Fig. 5:**
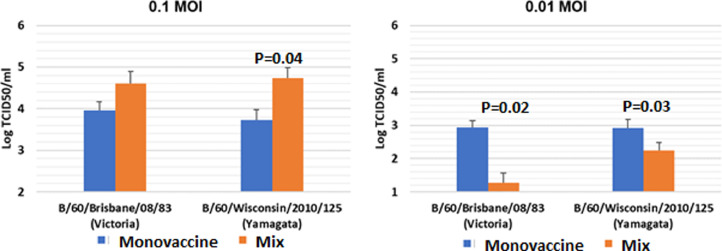
Evaluation of B/Victoria and B/Yamagata virus replication in MDCK cell line.
  The results obtained may indicate that the B/Victoria lineage is possibly more suppressed in the trivalent or quadrivalent composition as compared to V/Yamagata lineages. This must be taken into account in order to prevent a decrease in the infectious activity of monovalent B/Victoria-based vaccines when preparing trivalent or quadrivalent live influenza vaccines.

## Conclusions

Thus, it has been shown that the A/H1N1 pdm09 viruses was most suppressed in the trivalent vaccine. These data may explain the reduced immunogenicity of the A/H1N1 pdm09 component of the trivalent vaccine. During the randomized controlled trial of Russian-backbone LAIV it was demonstrated low replicative ability of A/HN1pdm09 components even in seronegative children. A/H3N2 and B viruses were shed at similar levels in 2017 and 2018 flu seasons [9]. Decreased shedding of H1 virus correlated with decreased production of hemagglutination-inhibition antibodies, in contrast to H3N2 and B viruses. When the 2009 A/H1N1 influenza pandemic emerged, the vaccine strain based on A/17/California/2009/38 (H1N1)pdm09 influenza virus was prepared and used for 10 years in the composition of trivalent and quadrivalent LAIVs It turned out that the pandemic vaccine strain showed reduced efficacy which has been associated with poor reproduction [10]. Although, a new vaccine strain based on A/Bolivia/559/2013(H1N1)pdm09 also demonstrated poor replication both in MDCK and human epithelium cell lines [11], and therefore was replaced by A/Slovenia/2903/2015(H1N1)pdm09-based vaccine strain. In this regard, the development of systems for the determination of infectious viral titers in multivalent vaccines is beneficial for timely assessment of LAIV vaccine strains.
