# Corrigendum to “Assessment of trivalent live influenza vaccines in MDCK cell line” [MethodsX 8 (2021) 101442]

**DOI:** 10.1016/j.mex.2021.101526

**Published:** 2021-09-21

**Authors:** G. Landgraf, Y.A. Desheva, L.G. Rudenko

**Affiliations:** a Federal State Budgetary Educational Institution of Higher Professional Education “St. Petersburg State University”, St. Petersburg, Russian Federation; b Federal State Budget Scientific Institution “Institute of Experimental Medicine”, St. Petersburg, Russian Federation

Incorrect Reference. In the original article, Table 1 (Page 2) was incorrectly written as

**Table:** 

Flu season	Serotype	Strain name	Virus titer (lоg10 EID50/0.5 ml)
2015-2016	H1N1 pdm09	A/17/ California /2009/38	7.4 ± 0.4
	H3N2	A/17/ Switzerland /2013/1	9.1 ± 0.5
	B	B/60/ Phuket /2013/26 (Yamagata)	8.9 ± 0.9
2017-2018	H1N1 pdm09	A/17/ New York /2015/5364	8.4 ± 0.1
	H3N2	A/17/ Hong Kong /2014/8296	8.0 ± 0.3
	B	B/60/ Brisbane /2008/83 (Victoria)	7.9 ± 0.3
2018-2019	H1N1 pdm09	A/17/ New York /2015/5364	8.2 ± 0.2
	H3N2	А/17/Singapore/2016/3571	8.1 ± 0.1
	В	В/60/Colorado/2017/1 (Victoria)	8.2 ± 0.3

We request to replace Table 1 (Page 2) with the following:

**Table:** 

Flu season	Serotype	Strain name	Name of the WHO recommended CVV strains	Virus titer (lоg10 EID50/0.5 ml)
2015-2016	H1N1 pdm09	A/17/ California /2009/38	A/California/7/2009	7.4 ± 0.4
	H3N2	A/17/ Switzerland /2013/1	A/Switzerland/9715293/2013	9.1 ± 0.5
	B	B/60/ Phuket /2013/26 (Yamagata)	B/Phuket/3073/2013	8.9 ± 0.9
2017-2018	H1N1 pdm09	A/17/ New York /2015/5364	A/Michigan/45/2015-like	8.4 ± 0.1
	H3N2	A/17/ Hong Kong /2014/8296	A/Hong Kong/4801/2014	8.0 ± 0.3
	B	B/60/ Brisbane /2008/83 (Victoria)	B/Brisbane/60/2008	7.9 ± 0.3
2018-2019	H1N1 pdm09	A/17/ New York /2015/5364	A/Michigan/45/2015-like	8.2 ± 0.2
	H3N2	А/17/Singapore/2016/3571	A/Singapore/INFIMH-16-0019/2016	8.1 ± 0.1
	В	В/60/Colorado/2017/1 (Victoria)	B/Colorado/06/2017	8.2 ± 0.3

ALSO In the original article, (Page 7) was incorrectly written as “Acknowledgements: This work was supported by a grant from the Russian Foundation for Basic Research “Molecular genetic markers of the safety and efficacy of multivalent vaccines against influenza and bacterial complications of influenza infection” ID 46175992.”

this reference was to be removed and replaced. We request to replace (Page 7) with the following: “Funding: The reported study was funded by RFBR - Russian Foundation for Basic Research, project number 19− 34-90021”.

The location of the citation should remain the same.

The Authors apologize for this error and state that this does not change the scientific conclusion of the article in any way.

